# Blood Pressure Affects the Early CT Perfusion Imaging in Patients with aSAH Reflecting Early Disturbed Autoregulation

**DOI:** 10.1007/s12028-023-01683-8

**Published:** 2023-02-17

**Authors:** Björn B. Hofmann, Daniel M. Donaldson, Igor Fischer, Cihat Karadag, Milad Neyazi, Guilherme S. Piedade, Yousef Abusabha, Sajjad Muhammad, Christian Rubbert, Daniel Hänggi, Kerim Beseoglu

**Affiliations:** 1 Department of Neurosurgery, Medical Faculty and University Hospital Düsseldorf, Heinrich-Heine-University Düsseldorf, Düsseldorf, Germany; 2 Department of Diagnostic and Interventional Radiology, Medical Faculty and University Hospital Düsseldorf, Heinrich-Heine-University Düsseldorf, Düsseldorf, Germany

**Keywords:** Aneurysmal subarachnoid hemorrhage, Early perfusion, Computed tomography, CT, Mean transit time, Blood pressure, Outcome

## Abstract

**Background:**

Early computed tomography perfusion (CTP) is frequently used to predict delayed cerebral ischemia following aneurysmatic subarachnoid hemorrhage (aSAH). However, the influence of blood pressure on CTP is currently controversial (HIMALAIA trial), which differs from our clinical observations. Therefore, we aimed to investigate the influence of blood pressure on early CTP imaging in patients with aSAH.

**Methods:**

We retrospectively analyzed the mean transit time (MTT) of early CTP imaging within 24 h after bleeding prior to aneurysm occlusion with respect to blood pressure shortly before or after the examination in 134 patients. We correlated the cerebral blood flow with the cerebral perfusion pressure in the case of patients with intracranial pressure measurement. We performed a subgroup analysis of good-grade (World Federation of Neurosurgical Societies [WFNS] I–III), poor-grade (WFNS IV–V), and solely WFNS grade V aSAH patients.

**Results:**

Mean arterial pressure (MAP) significantly correlated inversely with the mean MTT in early CTP imaging (*R* =  − 0.18, 95% confidence interval [CI] − 0.34 to − 0.01, *p* = 0.042). Lower mean blood pressure was significantly associated with a higher mean MTT. Subgroup analysis revealed an increasing inverse correlation when comparing WFNS I–III (*R* =  − 0.08, 95% CI − 0.31 to 0.16, *p* = 0.53) patients with WFNS IV–V (*R* =  − 0.2, 95% CI − 0.42 to 0.05, *p* = 0.12) patients, without reaching statistical significance. However, if only patients with WFNS V are considered, a significant and even stronger correlation between MAP and MTT (*R* =  − 0.4, 95% CI − 0.65 to 0.07, *p* = 0.02) is observed. In patients with intracranial pressure monitoring, a stronger dependency of cerebral blood flow on cerebral perfusion pressure is observed for poor-grade patients compared with good-grade patients.

**Conclusions:**

The inverse correlation between MAP and MTT in early CTP imaging, increasing with the severity of aSAH, suggests an increasing disturbance of cerebral autoregulation with the severity of early brain injury. Our results emphasize the importance of maintaining physiological blood pressure values in the early phase of aSAH and preventing hypotension, especially in patients with poor-grade aSAH.

**Supplementary Information:**

The online version contains supplementary material available at 10.1007/s12028-023-01683-8.

## Introduction

In the past years, research into aneurysmal subarachnoid hemorrhage (aSAH) has been focused on the phase of delayed brain ischemia (DCI), with the occurrence of vasospasm as the most prominent pathophysiological correlate [1]. Recently, it has been shown that early brain injury (EBI) is of great importance for the overall course of the disease [2]. Current studies indicate that the degree of cerebral hypoperfusion during the first hours after aSAH determines the severity of EBI, the occurrence of DCI, and the overall neurological outcome [2–7]. Several different perfusion imaging techniques and parameters have already been examined in the early evaluation of patients after bleeding, with computed tomography perfusion (CTP) imaging emerging as the most feasible technique [8–11]. As a result, CTP imaging is increasingly being used as a standard test during the initial and follow-up assessments of patients [12–14]. Particularly, the mean transit time (MTT) is a sensitive surrogate parameter to detect DCI-related perfusion impairment and is often used as a radiological follow-up parameter [8–11, 15–17]. In addition to its use as a follow-up parameter, the MTT of CTP imaging in the early phase after bleeding can be used as a predictor of DCI and neurologic outcome [6, 18–20]. Although an association between systemic blood pressure and cerebral perfusion in the light of altered cerebral autoregulation in EBI appears to be self-evident, robust data on the influence of blood pressure on the CTP in aSAH are lacking. In the DCI phase, so far only a trend for the cerebral blood flow (CBF) could be shown (e.g., in the HIMALAIA (Hypertension Induction in the Management of AneurysmaL subArachnoid haemorrhage with secondary IschaemiA) trial) [21, 22], but especially for the MTT, this influence is unclear, which differs with our clinical observations.

We hypothesize that the mean arterial pressure (MAP), possibly depending on the severity of SAH, correlates especially with the MTT in early perfusion imaging and that higher MAP is associated with shorter MTT, indicating better cerebral perfusion.

## Methods

All procedures were in accordance with the ethical standards of the institutional committee and with the 1964 Helsinki declaration and its later amendments. For this type of study, written formal consent was not required. The local ethics committee approved the study (study ID: 5760R). Data will be made available on reasonable request. We prepared the article according to the Strengthening the Reporting of Observational Studies in Epidemiology guidelines.

### Study Design and Study Population

A retrospective cohort study was performed for all patients with aSAH admitted to our tertiary neurovascular center from 2010 to 2015. The following inclusion criteria were applied: (1) SAH documented by an initial nonenhanced CT, (2) CTP imaging within 24 h after admission, and (3) documented blood pressure values within, at most, 60 min before or after the CT measurement. Patients were excluded from the study based on the following criteria: nonaneurysmal SAHs and unknown onset of aSAH (determined by self-reported time of the headache event or third-party report of sudden neurologic deterioration or unconsciousness). If a prolonged headache was present or the time of bleeding could not be determined with certainty, the patients were excluded from the analysis as well as patients with unevaluable early CTP imaging (e.g., due to severe motion artifacts), patients with pretreated aneurysms or recurrent SAH at the time of early CT imaging, patients with a history of previous cranial neurosurgical or endovascular intervention, and patients with recorded mean blood pressure < 30 mmHg or > 130 mmHg at the time of interest.

The remaining patients were initially dichotomized for subgroup analysis based on World Federation of Neurosurgical Societies (WFNS) grade (I–III vs. IV–V). Additionally, patients with WFNS grade V were evaluated separately to map only the most severe hemorrhages. For each patient, the average MTT was calculated from the early CT perfusion and analyzed for correlation with the recorded MAP value. According to the common model of cerebral autoregulation, further evaluation of CBF versus cerebral perfusion pressure (CPP) was performed for patients with external ventricular drainage (EVD) or intracranial pressure probe at the time of blood pressure recording.

### SAH Management

If an in-house native CT scan showed evidence of an aSAH, supplemental CT angiography and perfusion imaging were performed immediately in the same session. Patients in a peripheral hospital with evidence of SAH in a native CT scan and suspected aneurysm were transferred to our neurovascular center as soon as possible. They received CTP and CT angiography promptly on arrival. Treatment of patients with SAH in the early phase after hemorrhage was based on an in-house guideline following the applicable SAH guidelines [23]. In all patients, systolic blood pressure was maintained below 140 mm Hg before aneurysm occlusion. All patients with a Glasgow Coma Scale (GCS) ≤ 12 (patients with a poor WFNS grade) were intubated and ventilated before aneurysm repair, and the partial pressure of carbon dioxide was maintained between 30 and 35 mm Hg [11]. An EVD was implanted in intubated patients and patients with a poor WFNS grade [11]. Further standardized therapy was performed, as extensively described in earlier publications [11, 24]. Neurologic outcome was assessed by modified Rankin Scale (mRS) score at discharge and after 6 months.

### CT Perfusion Analysis

Computed tomography perfusion scans within 24 h after hemorrhage and before aneurysm repair were considered as early CTP imaging. CTP was performed as previously described: CTP data were acquired with a multislice CT scanner (SOMATOM Volume Zoom, Definition Flash or AS; Siemens Erlangen, Germany, 80 kV, 120 mAs, two adjacent slices, 10-mm slice thickness, one image over 35 s) [4, 6, 11, 25]. Three seconds after starting the CT, a contrast agent (30 ml, 400 mg iodine/ml) immediately followed by a saline chaser (30 ml), were injected with a flow rate of 5 ml/s. A peripheral intravenous line of ≤ 18 gauge in a cubital vein or a high-flow central venous catheter was required for contrast agent administration. Slices were positioned at the level of the central parts of lateral ventricles, parallel to a plane through the orbital floor and the external auditory meatus, thereby sampling the anterior, middle, and posterior cerebral artery territories, as well as the anterior and posterior border zones [26]. Calculation of the parameter images (MTT, CBF, among cerebral blood volume and time-to-maximum (Tmax)) was performed by using STROKETOOL-CT (Digital Image Solutions, Frechen Germany), which processes data using singular value decomposition of the arterial input function matrix. We used the software Angiotux CT 2D (ECCET 2006, Beck A. Aurich V.) for standardized parameter value extraction from the different cortical brain regions. A 10-mm wide band is automatically delineated along the cortex as a region of interest (ROI), omitting outer cerebrospinal fluid spaces, the rostral falx cerebri, and the superior sagittal sinus. The automated definition of the ROI was reviewed, and potential deviations were corrected as needed. A running average spanning 10° of the ROI was computed in 2° steps for each perfusion parameter, yielding 180 measurements per parameter per CTP scan.

### Blood Pressure Measurement

Vital parameters, such as blood pressure, are automatically documented and digitally stored with time stamps accurate to the second whenever a patient is connected to a stationary monitoring unit in either our emergency trauma room or neurosurgical intensive care unit. The blood pressure measurement is either a noninvasive measurement using an adequately sized blood pressure cuff on one of the upper arms or an invasive measurement from an arterial line (radial or brachial artery, occasionally femoral artery) with the transducer positioned at heart level. The emergency trauma room of our tertiary care facility is equipped with a CT scanner capable of perfusion imaging, which allows for brief time intervals between blood pressure measurements and CTP imaging in primarily admitted patients.

If the patient was primarily admitted to a peripheral hospital, the patient was directly transferred to our neurosurgical intensive care unit. The aforementioned system documents no values during patient transport to the CT. Blood pressure values recorded directly before or after transport were recorded and checked individually for plausibility. Of these two values, the single blood pressure value used for analysis was the one that was closer in time to the CTP imaging. If the pretransport and post-transport blood pressure values had the same time interval to CTP imaging, the pretransport values were preferred, as these were considered less susceptible to interference. Systolic, diastolic, MAP (MAP = diastolic + 1/3 [systolic-diastolic]), and CPP (if available) values with the shortest time interval to the CTP imaging were extracted from the database for evaluation.

### Statistical Analysis

Patients’ characteristics and clinical data were analyzed using RStudio, Version 1.3.1093 and Python Version 3.9.7. Our primary aim was to analyze the correlation of cerebral perfusion and blood pressure early after SAH. To achieve this, we calculated the average MTT over the whole cortex from the early CTP for each patient and correlated it with the recorded MAP by using the Pearson correlation coefficient, as the data are interval scaled. Normal distribution and the assumption of linearity were established by plotting the data in a histogram and scatter plot, respectively. Correlation between mean MTT and WFNS, Fisher score, and mRS was tested using the Kendall rank correlation coefficient because ordinal items in each scale were equal to or less than 6, according to Khamis et al. [27]. In the analysis of the 6-month mRS data, patients lost in the follow-up were excluded. Student’s *t*-test was used to compare the mean of two groups. The significance level was set to 0.05. Dependency between CPP and CBF was modeled using linear regression.

## Results

### Patients

From the initial 702 patients with SAH during the study period, a total of 568 were excluded due to various factors, including those with missing pretreatment early CTP imaging, unknown onset of SAH, known history of cranial neurosurgical intervention or, in most cases, lack of timely blood pressure recording in relation to the time of CTP imaging. Thus, after applying the inclusion and exclusion criteria, 134 patients were included in the analysis. Patients’ characteristics distinguished by subgroup are depicted in Table 1.

**Table 1:** Patients’ characteristics distinguished by subgroup

Parameter		Overall (*N* = 134)	WFNS I–III (*n* = 70)	WFNS IV–V (*n* = 64)	WFNS V (*n* = 34)
Age, mean (± SD) (yr)	55 (± 11)		53 (± 12)	58 (± 10)	56 (± 10)
Sex, *n* (%)					
Female	91 (68)		47 (67)	44 (69)	23 (68)
Male	43 (32)		23 (33)	20 (31)	11 (32)
Fisher grade, *n* (%)					
1	14 (10)		13 (19)	1 (2)	1 (3)
2	4 (3)		4 (6)	0 (0)	0 (0)
3	75 (56)		44 (63)	31 (48)	17 (50)
4	41 (31)		9 (13)	32 (50)	16 (47)
mRS discharge, *n* (%)					
0–2	54 (40)		45 (64)	9 (14)	4 (12)
3–5	56 (42)		21 (30)	35 (55)	17 (50)
6	24 (18)		4 (6)	20 (31)	13 (38)
mRS 6 months, *n* (%)					
0–2	74 (55)		50 (71)	24 (38)	11 (32)
3–5	27 (20)		11 (16)	16 (25)	7 (21)
6	24 (18)		4 (6)	20 (31)	14 (41)
Missing	9 (7)		5 (7)	4 (6)	2 (6)
Average MTT, mean (± SD) (ds)	40 (± 12)		38 (± 12)	42 (± 12)	40 (± 12)
MAP, mean (± SD) (mm Hg)	87 (± 21)		91 (± 19)	84 (± 18)	81 (± 18)

*ds* decisecond, *MAP* mean arterial pressure, *MTT* mean transit time, *mRS* modified Rankin scale, *SD* standard deviation, *WFNS* World Federation of Neurosurgical Societies

### Blood Pressure Data

**Fig. 1:**
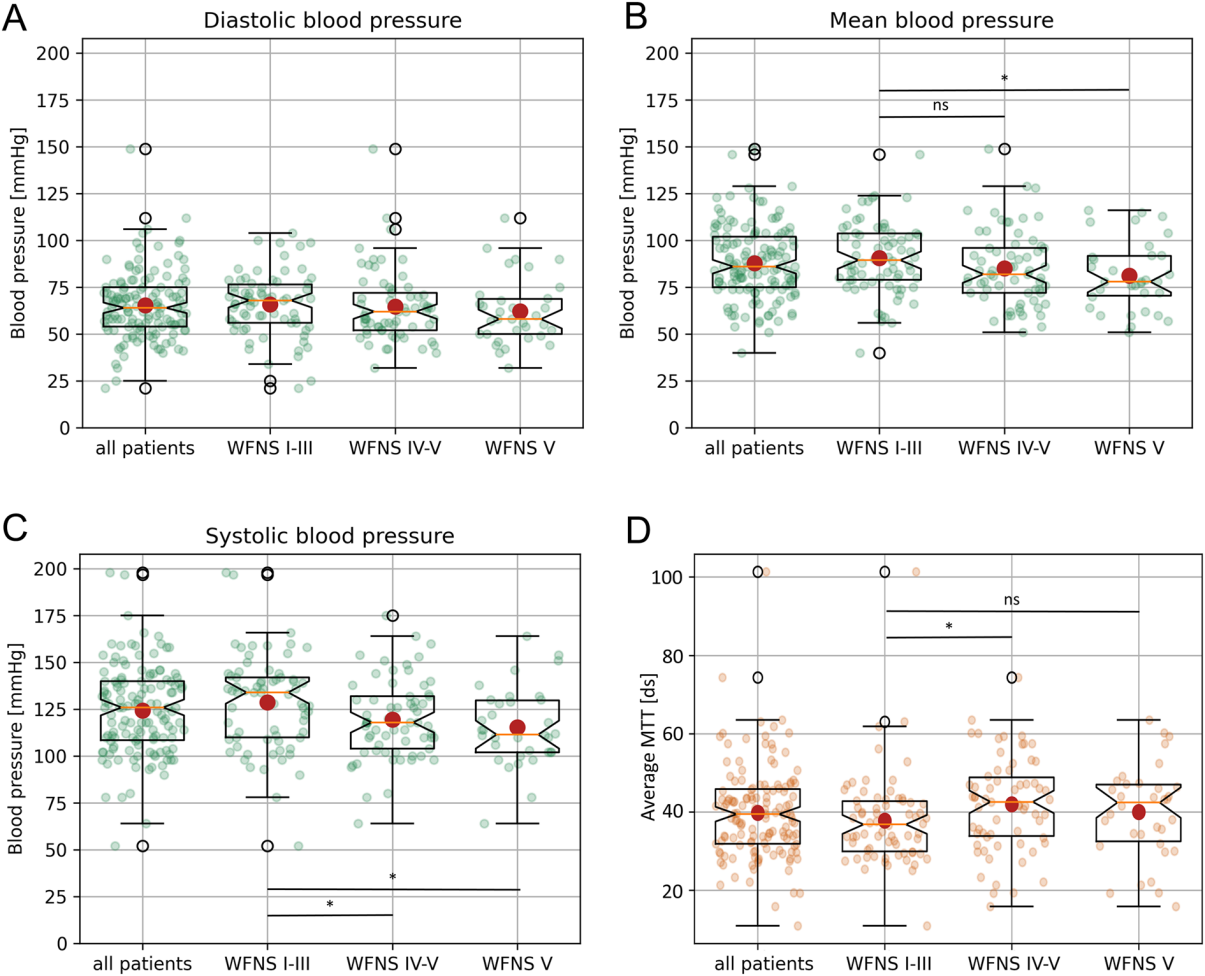
Comparison of mean, systolic, and diastolic blood pressure and MTT between patients with good-grade and poor-grade aSAH**.** The diastolic (**A**), mean (**B**), and systolic (**C**) blood pressure in mmHg for all patients, good-grade patients, poor-grade patients, and only WNFS grade V patients are depicted as mean ± SD. **D** Mean MTT of early CT perfusion imaging for all patients, good-grade patients, poor-grade patients, and only WNFS grade V patients are shown as mean ± SD (*p < 0.05). aSAH, aneurysmal subarachnoid hemorrhage, CT, computed tomography, ds, decisecond, MAP, mean arterial pressure, MTT, mean transit time, ns, not significant, SD, standard deviation, WNFS, World Federation of Neurosurgical Societies

The MAP was 87 mm Hg (± 21; systolic [s]: 124 ± 24; diastolic [d]): 65 ± 19) for all patients, 91 mm Hg (± 19; s: 129 ± 25; d: 66 ± 17) for patients with WFNS I–III, 84 mm Hg (± 22; s: 119 ± 21; d: 64 ± 20) for patients with WFNS IV–V and 81 mm Hg (± 18; s: 116 ± 21; d: 62 ± 18) in the subgroup including only patients with WFNS V. In the subgroup comparison, there was a significant difference for systolic (95% confidence interval [CI] 1.38 to 17.1, *p* = 0.02), but not for diastolic (95% CI − 4.3 to 8.5, *p* = 0.5) and mean blood pressure (95% CI − 0.54 to 13.4, *p* = 0.07) when comparing patients with WFNS I–III versus patients with WFNS IV–V (Fig. 1). The comparison of WFNS I–III versus WFNS V showed a significant difference in systolic (95% CI 3.8 to 22.5, *p* = 0.006) and mean (95% CI 1.6 to 17, *p* = 0.02), but not in diastolic blood pressure (95% CI − 3.6 to 11.1, *p* = 0.3; Fig. 1a).

The median time interval from the time of blood pressure measurement to CTP imaging was 9 min (mean = 5.8 ± 24 min) for all patients, 9 min (mean = 5.8 ± 22 min) for patients with WFNS I–III, 9 min (mean = 5.8 ± 25 min) for patients with WFNS IV–V, and 5.5 min (mean = 3.3 ± 23 min) for patients with WFNS V.

### MTT of Early CTP Imaging

The mean MTT was 4.0 s (± 1.2) for all patients, 3.8 s (± 1.2) for patients with WFNS I–III, 4.2 s (± 1.2) for patients with WFNS IV–V, and 4 s (± 1.2) for patients with WFNS V. The difference between patients with WFNS grade I–III vs. WFNS grade IV–V was significant (CI, − 8.3 to − 0.2; *p* = 0.04), whereas the difference between WFNS grade I–III patients versus WFNS V patients was not significant (CI, − 7.2 to 2.8; *p* = 0.4; Fig. 1b).

The MTT of early CTP in the whole study population significantly correlated with the initial WFNS ([T] = 0.17, *p* < 0.01), the mRS at discharge ([T] = 0.19 *p* < 0.01), and the mRS after 6 months ([T] = 0.17 *p* < 0.01), but not with the Fisher grade ([T] = 0.06 *p* = 0.37).

### Correlation Between MTT of Early CTP Imaging and MAP

**Fig. 2:**
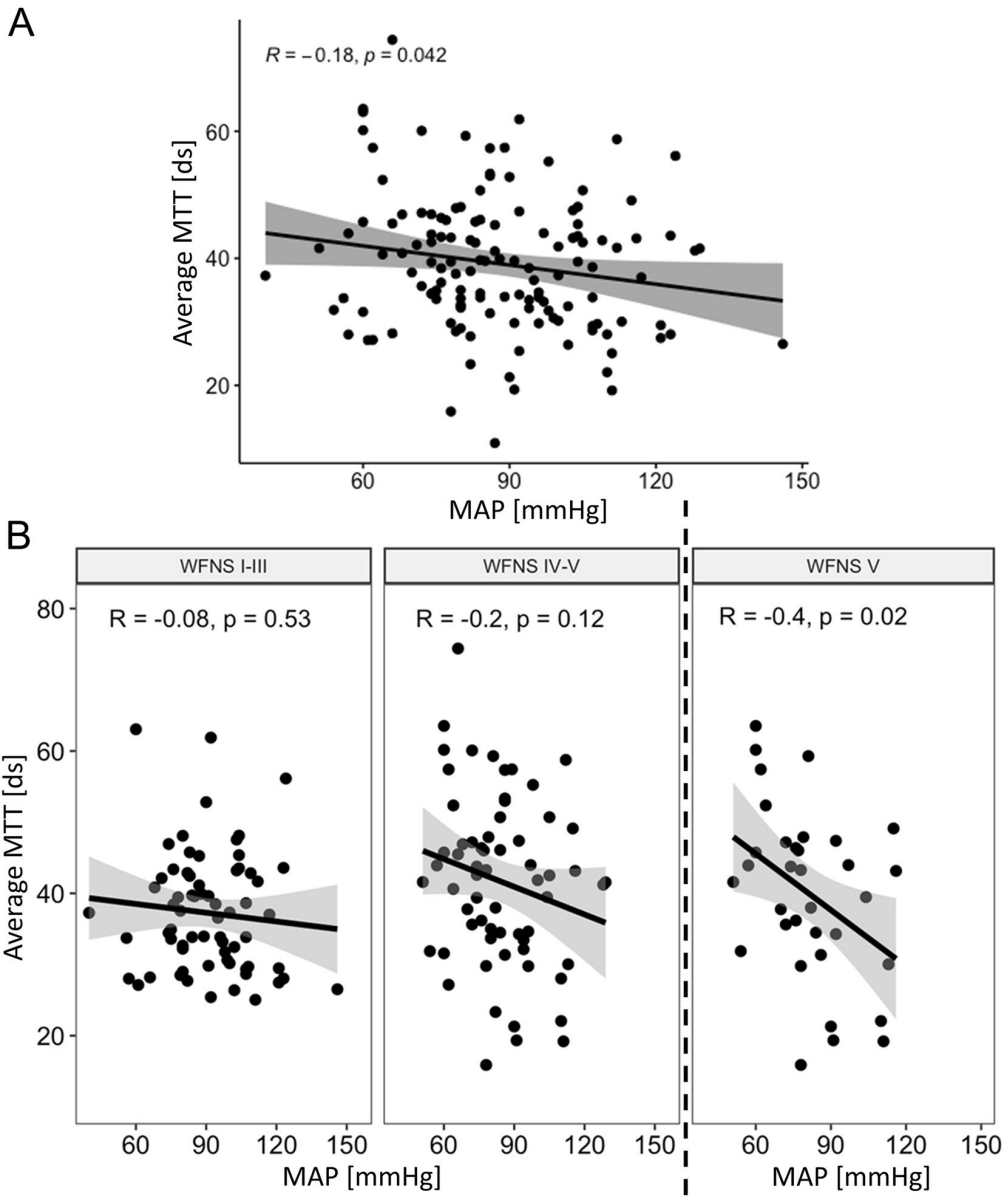
MTT correlates with blood pressure; the correlation increases with the severity of aSAH. **A** MTT of early CTP correlated significant with the MAP in the entire study population (*R* =  − 0.18, 95% CI − 0.34 to − 0.01, *p* = 0.042). **B** The dichotomized subgroups showed a nonsignificant correlation (*R* =  − 0.08, 95% CI − 0.31 to 0.16, *p* = 0.53; left panel) for the good WNFS grade patients and for the poor WFNS grade patients (*R* =  − 0.2, 95% CI − 0.42 to 0.05, *p* = 0.12; middle panel). However, when only patients with WFNS V are considered, there is a significant and strong correlation of MAP with MTT (*R* =  − 0.4, 95% CI − 0.65 to − 0.07, *p* = 0.02) (right panel). aSAH, aneurysmal subarachnoid hemorrhage, CI, confidence interval, CTP, computed tomography perfusion, ds, decisecond, MAP, mean arterial pressure, MTT, mean transit time, WNFS, World Federation of Neurosurgical Societies

The mean MTT in early CTP correlated significantly with the MAP in the entire study population (*R* =  − 0.18, 95% CI − 0.34 to 0.01, *p* = 0.042; Fig. 2a). Consequently, a lower MAP was associated with higher MTT. When the dichotomized WFNS subgroups were analyzed, a nonsignificant correlation for patients with WNFS I–III (*R* =  − 0.08, 95% CI − 0.31 to 0.16, *p* = 0.53) was observed (Fig. 2b, left panel). Likewise, a nonsignificant but stronger trend for patients with WFNS IV–V (*R* =  − 0.2, 95% CI − 0.42 to 0.05, *p* = 0.12) was observed (Fig. 2b, middle panel). However, if only patients with WFNS V are considered, there is a significant and strong correlation of MAP with MTT (*R* =  − 0.4, 95% CI − 0.65 to 0.07, *p* = 0.02; Fig. 2b, right panel).

### Correlation Between CBF of the Early CTP Imaging and CPP

**Fig. 3:**
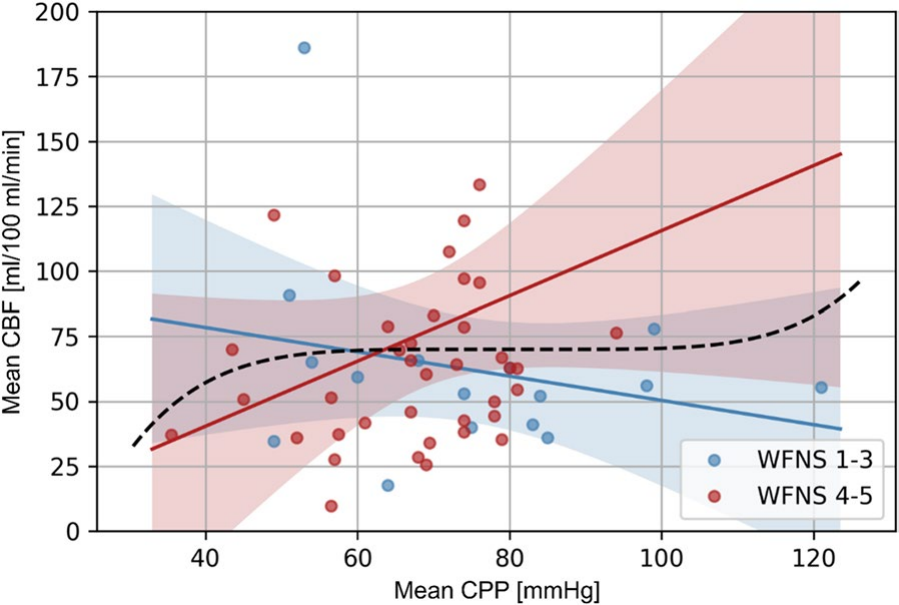
CBF tends to correlate with CPP in poor-grade patients. The CBF of early CTP did not correlate significantly with the CPP in the subgroup of patients with good WNFS grade aSAH (*n* = 16; *R* =  − 0.47, 95% CI − 1.5 to 0.6, *p* = 0.35; Fig. 3, blue). Poor WFNS grade patients (*n* = 40) showed a stronger, yet also nonsignificant, correlation of CBF with CPP (*R* = 1.25, 95% CI − 0.3 to 2.8, *p* = 0.12; Fig. 3, red). Outliers were excluded for better graphical presentation. aSAH, aneurysmal subarachnoid hemorrhage, CBF, cerebral blood flow, CI, confidence interval, CPP, cerebral perfusion pressure, CTP, computed tomography perfusion, WNFS, World Federation of Neurosurgical Societies (Color figure online)

When selecting patients with intracranial pressure measurement at the time of the documented blood pressure measurement, 16 good-grade patients and 40 poor-grade patients remained. The mean CBF in early CTP did not correlate significantly with the CPP in the subgroup of patients with good-grade SAH (*R* =  − 0.47, 95% CI − 1.5 to 0.6, *p* = 0.35; Fig. 3, blue). Poor-grade patients showed a stronger, yet also nonsignificant, correlation of CBF with CPP (*R* = 1.25, 95% CI =  − 0.3 to 2.8, *p* = 0.12; Fig. 3, red).

## Discussion

This retrospective study in patients with aSAH has three main findings:

1. MTT of early CTP inversely correlates with the MAP when analyzing the whole study population. A high MTT is associated with low mean blood pressure.
2. The more severe the aSAH, as measured by the WFNS grade, the stronger the inverse correlation of the MAP with the MTT.
3. There is a stronger correlation between CBF and CPP in patients with poor-grade aSAH than in patients with good-grade aSAH.

The MTT of CTP imaging is an important routine follow-up parameter in the monitoring of patients with aSAH. For example, in our neurovascular center, according to an in-house treatment protocol, if the MTT threshold of 41 deciseconds is exceeded, which, in our opinion, is a sign of a critical cerebral perfusion situation based on a large number of previous observations regarding MTT in patients with aSAH [25, 26, 28], a multimodal therapy is usually initiated. In addition, the MTT of CTP imaging is increasingly used in the phase of EBI as an early surrogate parameter of patient outcome [6, 15–18, 20, 29, 30]. Although an association between systemic blood pressure and cerebral perfusion in the light of altered cerebral autoregulation in EBI appears to be self-evident, robust data on this topic are lacking [21, 31]. Until this study, there has been no other study directly designed to investigate the association of blood pressure with CTP imaging in patients with aSAH. Based on our clinical observation, we hypothesized that the MAP correlates with the MTT of early CTP imaging, such that a lower MAP is associated with a longer MTT.

The results of the present study support our hypothesis, clearly showing an inverse correlation of MAP with MTT of early CTP in the EBI phase. This is already evident in the initial analysis of the entire study population (Fig. 2a). Although, in our view, the increasing inverse correlation with severity of aSAH is an even more striking observation (Fig. 2b). The inverse correlation increases in the study from patients with good-grade aSAH (WFNS I–III), with *R* =  − 0.08, to patients with poor-grade aSAH (WFNS IV–V), with *R* =  − 0.2 (Fig. 2b). However, this correlation does not reach the significance level. Hereby, we attribute the lack of significance of this observation to the reduced sample size caused by dichotomization. We assume that the inverse correlation would also become significant with a larger study population for these subgroups. In this context, it is even more impressive that a significant and even more substantial inverse correlation of MAP with MTT with *R* =  − 0.4 is found when only the patients with WFNS V are analyzed, resulting in an even smaller group size (Fig. 2b, right panel). Because, to our knowledge, the relationship between blood pressure and CTP imaging in the phase of EBI was not examined yet, our results can only be compared with observations related to the phase of DCI. The results of this study therefore extend the results of the prospective HIMALAIA (Hypertension Induction in the Management of AneurysmaL subArachnoid haemorrhage with secondary IschaemiA) trial, which could only show a nonsignificant effect of blood pressure on CBF of perfusion imaging in the DCI phase [21]. However, it should be noted that these results are only a side evaluation of a prospective trial aimed to compare the outcome of patients treated with and without induced hypertension after SAH [21, 22]. Furthermore, mainly the CBF of the CTP scans was the focus of this side evaluation [21]. Specific statements on the MTT, which, in our opinion, is of greater benefit in the monitoring of patients with aSAH, are not available [21]. It should also be noted that the study based its conclusion on data from only 13 patients with induced hypertension versus 12 patients without induced hypertension, as it was terminated prematurely because of difficult recruitment [21, 22].

Furthermore, the results of this study are well aligned with new insights into the pathophysiological processes after aSAH. Recent studies show that aSAH is followed by disturbed autoregulation affecting cerebral perfusion [32–36]. This has already been demonstrated several times in animal models and humans, in which the impairment of autoregulation has also been observed very early post bleeding in the phase of EBI [33–36]. Theoretically, impaired autoregulation is defined as the inability to maintain constant cerebral perfusion when changes in blood pressure occur [37, 38]. Consequently, a more powerful direct influence of blood pressure on cerebral perfusion is assumed. However, proof of the association of blood pressure with brain perfusion in CTP imaging has not been demonstrated in patients with aSAH until today. Our study adds relevant data to this discussion.

In our view, the results of this study underline the assumed effects of disturbed autoregulation on blood pressure. Blood pressure differences, which generally do not significantly affect cerebral perfusion, have an uninhibited, more direct influence on cerebral perfusion. Therefore, a lower blood pressure correlates with a higher MTT, i.e., a surrogate for poor cerebral perfusion. Furthermore, the results of the subgroup analysis also fit this theory. Our study shows that the relationship between MAP and MTT in early CTP imaging increases with the severity of aSAH, as measured by the WFNS grade, as already discussed above (Fig. 2). The WFNS as a neurological classification scale hereby most likely reflects the severity of the EBI and consequently the severity of disturbance of cerebral autoregulation. Therefore, in patients with poor-grade aSAH, changes in MAP lead more directly to changes in MTT. Particularly in the case of the most severely affected patients with aSAH, in whom intracranial circulatory arrest can occur as described before [39, 40], there may be parallels to the cerebral autoregulatory dysfunction observed after cardiac arrest [41, 42]. In contrast, patients with good-grade aSAH are less affected and display a less extensive EBI, reflected in a weaker correlation between the MAP and the MTT.

To support this theory of impaired autoregulation, we examined the common model of cerebral autoregulation and investigated the correlation of CBF with CPP in our patient cohort [38, 43, 44]. Even if the significance level was not reached in this analysis due to the even smaller sample size, it shows a stronger correlation for the poor-grade patients than the good-grade patients. This reflects the more severe disturbance of cerebral autoregulation in the poor-grade patients and supports our assumption.

In summary, the data from this study, for the first time, not only show the association of blood pressure with brain perfusion as measured by MTT in the early phase after SAH but also show the increase of dependence regarding the severity of hemorrhage measured by WFNS grade. Because numerous studies have already demonstrated that prolonged MTT in the EBI phase is associated with a poor outcome [6, 29], it can be deduced that initial blood pressure management is of high importance, especially for patients with poor-grade aSAH. This should by no means lead to induced hypertension in untreated aneurysms in the phase of EBI, but attention should be paid to maintaining physiological blood pressure values.

Some study limitations are worth noting:

1. Data were retrospectively collected, and only patients with early CTP were included. It is unclear whether there was a selection bias in the choice of patients who received CTP imaging prior to the aneurysm repair. Nevertheless, the study population is relatively large for a single-center study. It is overall well-balanced, for example, regarding patients with good-grade and poor-grade SAH according to their WFNS score. We believe it is unlikely that our inclusion/exclusion criteria introduced a relevant bias.
2. Because of technical constraints, the blood pressure values are measured with a certain time offset to the early CTP imaging. Therefore, no digitally documented value is available from the exact imaging time. Especially for patients primarily admitted to an external hospital and then directly transferred to our intensive care unit, blood pressure values during transport and imaging are not recorded digitally. This results in a blind spot at the time of CTP imaging. This is one major limitation of this study. Yet, we aim for stable blood pressure without blood pressure peaks or dips during treatment. We therefore consider the blood pressure values recorded shortly before or after imaging to be representative. For subsequent prospective studies, however, a purely arterial blood pressure measurement with a recording of the blood pressure at the imaging time should be performed. The proven possibility of minimal variations in the CPP values due to changes in the patient’s position (30° torso elevation vs. supine in the CT) should also be taken into account, although in our study, this positional change most likely affects all patients to the same extent [45]. Furthermore, influences on the results of this study by external factors such as stimulation of patients on transport to the CT scanner affecting MAP and intracranial pressure should also be considered.
3. Patients with an inserted EVD are, in most cases, sedated and often also ventilated. The influence of sedation on the result of this study cannot be ruled out. Yet, an influence of the intracranial pressure on the results of the study is considered unlikely. Patients without EVD are conscious and a normal intracranial pressure can therefore be assumed, and in patients with EVD the intracranial pressure is normalized to a physiological level. Further subgroup analysis of dichotomized subgroups of patients with versus without EVD at the imaging time is attached in the supplementary material.
4. Data from CTP imaging are difficult to compare one-to-one between different setups due to different CTP protocols and postprocessing. In addition, diseases affecting the microvasculature such as atheriosclerosis, hypertension, or diabetes may influence the MTT. Additionally, metabolic state, use of sedatives, and concomitant medication may potentially impact the MTT, as well. Therefore, the effect of these factors on the results of this study remains unclear. Furthermore, it is unclear whether there are differences in the MTT when blood pressure values are present spontaneously or are influenced by medication. Similarly, it is not clear whether mean MTT alone or a combination of different parameters of CTP best represents the relevant pathophysiological changes in patients with aSAH, which should be taken into account when interpreting the data.

## Conclusions

In the present cohort of 134 patients with aSAH, MTT of early CTP inversely correlates with the MAP, indicating early impairment of cerebral autoregulation in the phase of EBI. A low MAP is associated with a prolonged MTT. The subgroup analysis shows that the inverse correlation becomes stronger with the severity of aSAH as measured by the WFNS, whereas, in particular, WFNS grade V patients demonstrated a significant, strong, inverse correlation of MAP and MTT.

These results emphasize the importance of maintaining physiological blood pressure values in the EBI phase and preventing hypotension, especially in patients with poor-grade aSAH. Further prospective studies should seek to confirm this influence of MAP on MTT in EBI and should verify whether the early blood pressure values correlate with the outcome of the patients post aSAH.

## Supplementary Information

Below is the link to the electronic supplementary material.

Supplementary file1 (PDF 210 KB)

## Abbreviations

aSAH: Aneurysmal subarachnoid hemorrhage
CBF: Cerebral blood flow
CPP: Cerebral perfusion pressure
CTA: Computed tomography angiography
CTP: Computed tomography perfusion, DCI: delayed cerebral ischemia
EBI: Early brain injury
EVD: External ventricular drainage
ICP: Intracranial pressure
MAP: Mean Arterial Pressure
MRI: Magnetic Resonance Imaging
mRS: Modified Rankin Scale
MTT: Mean Transit Time
RA: Resuscitation Area
SVD: Singular value decomposition
