# Supplementary material for: Blood Pressure Affects the Early CT Perfusion Imaging in Patients with aSAH Reflecting Early Disturbed Autoregulation
Source: Neurocrit Care. 2023 Feb 17;39(1):125–34. doi: 10.1007/s12028-023-01683-8 (PMC10499698; doi:10.1007/s12028-023-01683-8)
Supplement: Supplementary file 1 — Supplementary file1 (PDF 210 KB) [file 12028_2023_1683_MOESM1_ESM.pdf]

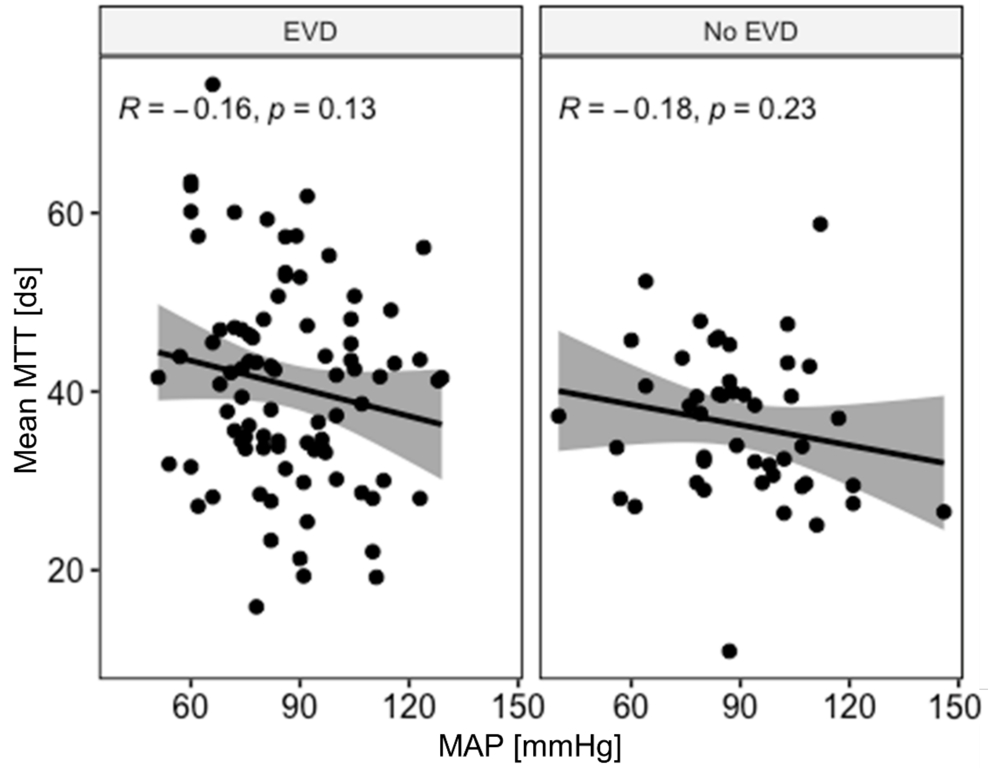

Supplemental Figure: Subgroup analysis for patients with vs. without EVD.

The dichotomized subgroups showed a non-significant correlation (R = -0.16, 95% CI = -0.36-0.05, p = 0.13; left panel) for patients with EVD and for patients without EVD (R = -0.18, 95% CI = -0.44-0.12, p = 0.23; right panel). MAP = mean arterial pressure, ds = decisecond, MTT = mean transit time, R = Regression coefficient.
